# Antifungal Susceptibility Testing: Current Role from the Clinical Laboratory Perspective

**DOI:** 10.4084/MJHID.2014.030

**Published:** 2014-04-07

**Authors:** Brunella Posteraro, Riccardo Torelli, Elena De Carolis, Patrizia Posteraro, Maurizio Sanguinetti

**Affiliations:** 1 Institute of Public Health (Section of Hygiene), Università Cattolica del Sacro Cuore, Largo F. Vito, 1-00168, Rome, Italy.; 2 Institute of Microbiology, Università Cattolica del Sacro Cuore, Largo F. Vito, 1-00168, Rome, Italy.; 3 Clinical Laboratory, Ospedale San Carlo, Via Aurelia, 275-00165, Rome, Italy.

## Abstract

Despite availability of many antifungal agents, antifungal clinical resistance occurs, perhaps as a consequence of an infecting organism found to be resistant in vitro to one or more antifungals tested. From what derives the important current role of the in vitro antifungal susceptibility testing (AFST), that is to determine which agents are like to be scarcely effective for a given infection. Thus, AFST results, if timely generated by the clinical microbiology laboratory and communicated to clinicians, can aid them in the therapeutic decision making, especially for difficult-to-treat invasive candidiasis and aspergillosis. Although recently refined AFST methods are commercially available for allowing a close antifungal resistance surveillance in many clinical setting, novel assays such as flow cytometry or MALDI-TOF mass spectrometry are upcoming tools for AFST. Based on short-time antifungal drug exposure of fungal isolates, these assays could provide a reliable means for quicker and sensitive assessment of AFST.

## Introduction

Although several factors are key determinants of antifungal clinical resistance,^1^ which is referred to as the persistence or progression of a fungal infection despite the administration of appropriate antifungal therapy, there is a general consensus that clinical outcomes are better when treatments are started early.^2^,^3^ Almost all the classes of systemically active antifungal agents available to date, such as polyenes (i.e., amphotericin B), azoles, flucytosine, and the newest echinocandins contribute to improve the management of invasive fungal infections (IFIs).^4^–^6^ Nevertheless, the rate of antifungal failures is high, and the emergence of resistant fungal strains is a growing concern, particularly for strains capable of exhibiting resistance to commonly prescribed antifungal drugs.^7^ Eighteen (11.1%) of 162 fluconazole-resistant bloodstream isolates of *Candida glabrata* collected during two large surveillance programs were found to be cross-resistant to one or more of the echinocandins.^8^ Likewise, patients with chronic pulmonary *Aspergillus* infection who receive prolonged (tri)azole therapy are at risk of resistant aspergillosis,^9^ with an evolving spectrum of resistance owing to the emergence of non-*cyp51A*-mediated mechanisms,^10^ as well as are at risk azole-naïve patients due to the presence of resistant TR/L98H strains (i.e., carrying a substitution at codon 98 in the *cyp51A* gene in combination with a 34 base-pair tandem repeat in the gene promoter) in the environment.^11^,^12^ Thus, while two-thirds of surveyed Dutch patients with azole-resistant *Aspergillus* disease had not history of previous azole exposure (with all *A. fumigatus* isolates from patients with invasive aspergillosis harboring the TR/L98H mutation),^13^ recent epidemiological data show that this resistance mechanism, first emerged in the Netherlands, is expanding not only in European countries but also in China, Iran, and India.^10^

## Antifungal Susceptibility Testing to Aid the Management of IFI Patients

The primary utility of antifungal susceptibility testing (AFST) arises from the concept that susceptibility (or resistance) to an antifungal agent selected for the therapy would allow some prediction about the impact that administration of the agent tested in vitro has on the clinical outcome of infection caused by the treated organism.^14^,^15^ Therefore, clinical microbiologists are currently engaged to determine the growth of fungi under different drug concentrations so as to yield the minimum inhibitory concentration (MIC) for a specific infecting isolate, that is an in vitro measure of susceptibility (expressed as growth inhibition) which helps to predict the therapeutic efficacy.^16^ Thus, it is important that MIC results are timely communicated to physicians to guide them in the therapeutic decision making, in the same way that antibacterial testing aids in the clinical guidance of bacterial infections.^17^

As attested by several studies evaluating the role of “real-time” AFST in managing patients with invasive *Candida* infections,^18^ physicians frequently (and appropriately) adjust the therapy on the basis of MIC results, although a clearly defined association between the timely receipt of antifungal therapy and poor outcome after *Candida* bloodstream infection due to a resistant isolate is lacking to date.^2^ Indeed, Collins et al.^19^ reported that the susceptibility testing (especially when done in-house) of *C. glabrata* isolates may facilitate quicker interventions (i.e., de-escalation of therapy from an expensive echinocandin to fluconazole) for patients with documented *C. glabrata* fungemia, thereby resulting in lower overall treatment costs. Likewise, Grim et al.^20^ found that receipt of appropriate early antifungal therapy (i.e., administered within 72 h of a positive culture being drawn) was associated with a significant (*P* = 0.047) survival benefit for patients who were effectively treated for ≥24 h, and their results were supported by the inclusion of routine AFST to optimally assess the adequateness of therapy.

Unlike *Candida* infection, there is only a limited number of reported *Aspergillus* infection cases that could elucidate the clinical impact of azole resistance on the patient’s outcome,^21^ and this situation has hindered the wide application of in vitro AFST of *Aspergillus* species. However, in an attempt to establish clinically derived breakpoints for *Aspergilli* that would help physicians to interpret the MIC values as produced from the clinical microbiology laboratory, a pragmatic (and not formal) approach was followed by Verweij et al.^21^ Thus, taking MIC distribution, pharmacokinetic/pharmacodynamic parameters of antifungal azoles, in vivo experimental correlation between *cyp51A* point mutations and failure, and clinical experience into account, interpretive breakpoints were proposed, that is MICs >2 μg/ml for itraconazole and voriconazole and >0.5 μg/ml for posaconazole.^21^ These breakpoints were able to discriminate between wild-type (that refers to isolates without mutational or acquired mechanisms of resistance) and non-wild-type (that refers to isolates with mutational or acquired mechanisms of resistance) MIC distributions for itraconazole and voriconazole among 325 consecutive clinical *A. fumigatus* isolates from the Nijmegen fungus culture collection.^21^ Based on these findings, a 4-well azole-agar dilution (4D) plate (i.e., 3 wells were each containing one of azoles: itraconazole 4 μg/ml, voriconazole 1 μg/ml, or posaconazole 0.5 μg/ml; and the fourth azole-free well served as control growth) was developed as a screening test for identifying potentially resistant *A. fumigatus* isolates.^22^ In parallel, Pfaller et al.^23^ used a collection of 637 geographically diverse, clinical isolates of *A. fumigatus* tested against itraconazole, posaconazole, and voriconazole, to assess the wild-type MIC distribution and epidemiological cutoff values (ECVs), that is MIC threshold values for differentiating wild-type isolates from non-wild-type isolates, for *A. fumigatus* and the mold-active triazoles.

By contrast, due to scarce (and less frequent than for azoles) tendency to carrying out AFST for *Aspergillus* isolates,^24^ perhaps as a result of technical difficulties and suboptimal reproducibility of the methods employed,^25^ echinocandin resistance in *Aspergillus* species is much less known.^22^ Although the caspofungin is recommended as a second line treatment choice for invasive aspergillosis^26^, and often administered in combination with amphotericin B,^21^, however, breakthrough infections (though sporadic) have been reported in patients under caspofungin therapy,^27^–^29^, and they involved *A. fumigatus* isolates, with elevated minimum effective concentrations (MECs) to caspofungin. The MEC endpoint, defined as the lowest drug concentration that leads to the growth of small, rounded, compact hyphal forms as compared to the hyphal growth seen in the growth control well, was suggested for testing antifungal susceptibility of *Aspergilli* to echinocandins, rather than the MIC;^25^ nonetheless, MEC remains technically difficult to determine.

## Antifungal Susceptibility Testing in the Daily Laboratory Practice

Several recommendations for routine use of AFST of *Candida* species in the clinical microbiology laboratory have been developed.^18^ They include testing of fluconazole and an echinocandin against *C. glabrata* isolated from deep sites and, possibly, against other species of *Candida*, unless their antifungal susceptibility pattern is predictable (i.e., for *Candida krusei*); use of clinical breakpoints (CBPs) or ECVs to interpret MIC values as appropriate; considering cross-resistance between fluconazole and all other triazoles (itraconazole, posaconazole, and voriconazole) to be complete for *C. glabrata*; and careful choice of susceptibility testing methods.^18^ In essence, a selective application of AFST, together with a precise identification of *Candida* to the species level,^30^ should be useful in selecting agents for primary therapy as well as in a de-escalating strategy,^18^ especially in difficult-to-manage cases of invasive candidiasis.^31^

With regards to *Aspergillus* species, it is currently recommended to perform AFST of clinically relevant *Aspergilli* (with isolates at least identified to the species level)^32^ as an adjunct to the treatment for IFI patients when therapeutic failure of initial therapy or breakthrough infection occur, and for patients with disease and long-term triazole treatment and/or recurrent isolation of an *Aspergillus* species.^25^ Also, whereas isolates of *Aspergillus* species known to be intrinsically drug-resistant (e.g., *A. terreus* against amphotericin B) need to be not usually tested,^25^ MIC determination could be useful to monitor the emergence of polyene resistance in *Aspergillus* species such as *A. flavus*.^33^

## Conventional and Novel Laboratory Assays for Testing Antifungal Susceptibility

Standardized microdilution-based procedures by the Clinical and Laboratory Standards Institute (CLSI) and the European Committee on Antibiotic Susceptibility Testing (EUCAST),^34^–^38^ are universally accepted for performing AFST (Table 1), but these procedures are complex, time-consuming, and not intended for routine use.^39^ As a result of a multistep process based on the analysis of MIC distribution curves for wild-type populations and the clinical relationship between MIC values and efficacy,^40^,^41^ CLSI/EUCAST MIC breakpoints (i.e., obtained with CLSI/EUCAST reference methods in specialized mycology laboratories) are to date available to interpret the AFST results of amphotericin B, azoles, and echinocandins for *Candida*, and amphotericin B and azoles for *Aspergillus*.^42^ Besides to be an important step in establishing fungal CBPs, the MIC distributions of wild-type fungal populations provide a measure of the ECVs, which, in the absence of specific CBPs, may be very useful in antifungal resistance surveillance to monitor the emergence of resistant isolates (i.e., those with gene mutations associated with reduced therapeutic responses).^7^ Also, AFST using the CLSI/EUCAST reference methods is a precious tool for studying the in vitro activity of new and experimental compounds, as well as the epidemiology of antifungal-resistant fungi. Finally, through recently refined AFST methods,^24^ coupled with detection of molecular fungal alterations conferring reduced antifungal drug susceptibility,^43^ often directly from clinical specimens,^44^,^45^ it is now possible to ensure a close antifungal resistance surveillance in many clinical settings. The detection of *cyp51A* gene mutations in primary clinical specimens is still the sole strategy for detecting *Aspergillus* resistance to triazoles in the absence of culture confirmation, which occurs in most cases of invasive and chronic pulmonary aspergillosis, making an MIC determination impossible.^21^,^22^ However, these nucleic acid-based assays, though permitting quicker detection of azole-resistance in culture positive samples, are to date not standardized or practical for most clinical laboratories,^42^ in addition to be unable to reveal the influence from other resistance mechanisms.^21^,^22^ Given these concerns and the aforementioned increasing number of resistance cases, performing susceptibility testing of *Aspergillus* isolates before and during antifungal treatment can be clinically relevant.^22^ Yet, since obtaining repeated *Aspergillus* positive cultures from patients receiving antifungal therapy (that would allow to prove that a treatment failure is actually due to an antifungal-resistant organism) is an uncommon clinical scenario, monitoring of the galactomannan (GM) biomarker through serial GM index measurements following antifungal treatment^46^,^47^ could be effective for detecting resistance to antifungal therapy.

Commercially available tests, such as Sensititre YeastOne, Etest, and the fully automated Vitek 2 yeast susceptibility system (Table 1), all easy-to-use modifications from the CLSI/EUCAST reference methods are widely used for testing antifungal susceptibility of relevant *Candida* and *Aspergillus* species.^7^ While the commercial tests show a good essential agreement (defined as MICs within 2 dilutions) with the reference methods, the categorical agreement (i.e., agreement in the categorization of an isolate as susceptible, intermediate, or resistant) may be lower, especially for the echinocandin class of antifungal agents.^48^–^50^ Thus, it was noted that clinical fungal isolates should not be classified as resistant in vitro by commercial methods, unless standardization processes and setting of their own breakpoints have been undertaken.^39^ As MIC determination by reference methods is highly recommended for patient management,^51^ periodical epidemiological surveys of deep, blood, and mucosal infections should be done to monitor antifungal susceptibilities of *Candida* and *Aspergillus*. So, local surveillance MIC data, derived from a routine microbiology laboratory workflow, can be used to develop treatment strategies, particularly by clinicians who prescribe preemptively or empirically antifungals in hematology, transplantation, or intensive care units. In parallel, antifungal resistance surveillance studies should also investigate air samples for the presence of *A. fumigatus* resistant to medical triazoles in the hospital environment to ascertain the local resistance risk among filamentous fungi. Therefore, both clinical and environmental samples can be screened using the aforementioned 4D plates^52^ to evaluate to what extent exposure to azoles in patients^9^ or in the environment^11^ contributes to antifungal resistance in the hospital setting.

New diagnostic approaches, based on emerging technologies such as flow cytometry (FC), MALDI-TOF mass spectrometry (MALDI-TOF MS), and isothermal microcalorimetry (IMC) (Table 1), have been developed to expand, and potentially improve, the capability of the clinical microbiology laboratory to yield AFST results. By flow cytometry (FC), the effects of a given antifungal drug can be appreciated by observing alterations in the fungal cell viability (rather than the growth inhibition as in conventional methods) that will be identified via changes in the measured cell fluorescence;^53^ this led to assess the minimum fluorescence-enhancing concentration (MFEC), that is the lowest concentration of antifungal agent to which the percentage of cells showing altered fluorescence is superior to a predetermined cutoff value (set at 50% for *C. glabrata* and *C. krusei*, and at 40% for *Candida parapsilosis*).^54^ Using MALDI-TOF MS, a simple and rapid AFST assay (named ms-AFST) was established to discriminate susceptible and resistant isolates of *Candida albicans* after a 3-h incubation in the presence of “breakpoint” concentrations of caspofungin; after the fungal spectra at concentration 0, 0.03, or 32 μg/ml of caspofungin were compared to create individual composite correlation index (CCI) matrices, the tested isolates were classified as susceptible or resistant to caspofungin if the CCI values of the spectra at 0.03 and 32 μg/ml were, respectively, higher or lower than the CCI values of the spectra at 0.03 and 0 μg/ml.^55^ Finally, IMC was evaluated for “real-time” susceptibility testing of *Aspergillus* species, by measuring the thermal variations induced by the action of antifungals; this led to define the minimal heat inhibitory concentration (MHIC), that is the lowest antifungal concentration which inhibits 50% of the total heat produced by the growth control at 48 h or, only for anidulafungin and caspofungin, the lowest antifungal concentration which reduces the heat-flow peak by 50%.^56^ It should be noted that while the time-to-result of an IMC assay is surely not different from that of conventional MIC methods (Table 1), the susceptibility endpoints for the echinocandins are hard to determine due to significant trailing growth, and the MEC reading is actually subjective and poorly reproducible.^22^ As an alternative to the classical MIC, the new endpoints could then provide a simple, reliable, and accurate means of identifying antifungal-resistant isolates, thus potentiating the practicability and the clinical utility of AFST. However, further studies need to be undertaken to improve reproducibility and standardization of the recent developments in AFST, in order to transform them in clinical useful assays in the next future.

## Conclusions

Although AFST is considered currently a valid method, it remains a very dynamic field of clinical microbiology, as further research is needed before MICs are independently used to guide treatment decisions^15^ and before the standardization process is completed to include all known antifungal compounds and fungal species^42^. While a crucial issue is whether current AFST methods and antifungal breakpoints are capable of identifying resistant fungal isolates, associated with treatment failures, new alternate AFST methods should be introduced to improve the detection of antifungal resistance, which is perhaps the most challenging goal in clinical microbiology.

## Figures and Tables

**Table 1:** Reference and non-reference methods for antifungal susceptibility testing of *Candida* and *Aspergillus* clinical isolates^a^

Characteristic	Standardized Methods		Commercial Methods			Novel Methods			

	CLSI	EUCAST	SYO	Etest	Vitek 2	FC	MALDI-TOF MS	IMC	4D plate
Suitability	Yeasts (M27-A3), molds (M38-A2)	Fermentative yeasts (EDef.7.2), molds (EDef.9.1)	Yeasts and molds	Yeasts and molds	Yeasts	*Candida* species	*Candida albicans*	*Aspergillus* species	*Aspergillus* species
Format^b^	BMD	BMD	BMD	Agar-based method	BMD (AST-YS06 cards)	Broth dilution	Broth dilution	Broth dilution	Agar dilution
Temperature	35 °C	35–37 °C	35–37 °C	35–37 °C	Instrument incubator	35 °C	37 °C	37 °C	37 °C
Incubation time	24–48 h	24–48 h	24–48 h	24–48 h	12–24 h	1–4 h	3 h	48 h	48 h
Reading	Visually	Visually/spectrophotometrically	Visually	Visually	Automatically	Fluorescence microscopy	Mass spectrometry	Isothermal microcalorimeter	Visually
Endpoint^c^	MIC, MEC (only for echinocandins)	MIC	MIC, MEC (only for echinocandins)	MIC	MIC	MFEC	CCI-measured spectral comparison	MHIC	No growth
Use (pros and cons)^d^	Detecting resistant isolates, but restricted to specialized laboratories	Detecting resistant isolates, but restricted to specialized laboratories	Routine testing of isolates, but categorization of resistant isolates not advised	Routine testing of isolates, but categorization of resistant isolates not advised	Routine testing of isolates, but categorization of resistant isolates not advised	Rapid detection of antifungal resistance, but today not applied to the routine clinical practice	Rapid detection of caspofungin resistance, but today not applied to the routine clinical practice	Potential detection of resistant isolates, but still in an infancy stage	Screening for potentially azole-resistant isolates, but confirmation by the reference method required

a Details about the reference (CLSI and EUCAST) and non-reference (commercial and novel) methods are given in the text. CLSI, Clinical and Laboratory Standards Institute; EUCAST, European Committee on Antimicrobial Susceptibility Testing; SYO, Sentitre YeastOne; FC, flow cytometry; MS, mass spectrometry; IMC, isothermal microcalorimetry; 4D; 4-well azole-agar dilution.

b The indicated commercial methods actually represent modifications of standardized agar or broth microdilution (BMD) methods.

c The indicated endpoints include MFEC (minimum fluorescence-enhancing concentration) and MHIC (minimal heat inhibitory concentration) for the FC and IMC methods, respectively; for MALDI-TOF MS, the endpoint represents a composite correlation index (CCI) value as obtained after the CCI values, derived by matching fungal spectra at established caspofungin concentrations, were calculated; for the 4D plate method, the endpoint is expressed as the absence of growth onto azole-containing agar as compared to the free-azole growth control well. These endpoints are alternatives to the traditional MIC (minimum inhibitory concentration) and MEC (minimum effective concentration) used with conventional methods, as indicated.

d According to expert recommendations on the use of each method in the clinical microbiology laboratory, as specified in the text.
